# Content and quality of physical activity ontologies: a systematic review

**DOI:** 10.1186/s12966-023-01428-y

**Published:** 2023-03-13

**Authors:** Maya Braun, Stéphanie Carlier, Femke De Backere, Annick De Paepe, Marie Van De Velde, Delfien Van Dyck, Marta M. Marques, Filip De Turck, Geert Crombez

**Affiliations:** 1 Department of Experimental Clinical and Health Psychology, Ghent University, Ghent, Belgium; 2 IDLab, Department of Information Technology, Ghent University – imec, Ghent, Belgium; 3 Department of Movement and Sports Sciences, Ghent University, Ghent, Belgium; 4 Nova Medical School, Comprehensive Health Research Centre (CHRC), NOVA University of Lisbon, Lisbon, Portugal

**Keywords:** Ontology, Physical activity, Classification, Quality assessment, Systematic review

## Abstract

**Introduction:**

Ontologies *are a formal way* to represent knowledge in a particular *field and* have the potential to transform the field of health promotion and digital interventions. However, few researchers in physical activity (PA) are familiar with ontologies, and the field can be difficult to navigate.

This systematic review aims to (1) identify ontologies in the field of PA, (2) assess their content and (3) assess their quality.

**Methods:**

Databases were searched for ontologies on PA. Ontologies were included if they described PA or sedentary behavior, and were available in English *language*. We coded whether ontologies covered the user profile, activity, or context domain. For the assessment of quality, we used 12 criteria informed by the Open Biological and Biomedical Ontology (OBO) Foundry principles of good ontology practice.

**Results:**

Twenty-eight ontologies met the inclusion criteria. All ontologies covered PA, and 19 included information on the user profile. Context was covered by 17 ontologies (physical context, *n* = 12; temporal context, *n* = 14; social context: *n* = 5). Ontologies met an average of 4.3 out of 12 quality criteria. No ontology met all quality criteria.

**Discussion:**

This review did not identify a single comprehensive ontology of PA that allowed reuse. Nonetheless, several ontologies may serve as a good starting point for the promotion of PA. We provide several recommendations about the identification, evaluation, and adaptation of ontologies for their further development and use.

**Supplementary Information:**

The online version contains supplementary material available at 10.1186/s12966-023-01428-y.

## Background

The idea of ontologies can be traced back to early philosophers studying how to describe and categorize ‘what is’ in the world. Ontologies refer to the various ways to structure and classify our knowledge about the world, including typologies or taxonomies such as the periodic table of Mendeleev, the Linnaean classification system of plants, the compendium of physical activities [1], or the taxonomy of behaviour change technique [2]. Within computer and information sciences, the term ‘ontologies’ has become reserved for formal classification systems that are computer-readable, provide clear definitions of concepts (classes) and their properties, and allow formal modelling of both simple and complex relationships between concepts [3, 4]*.*

**Fig. 1:**
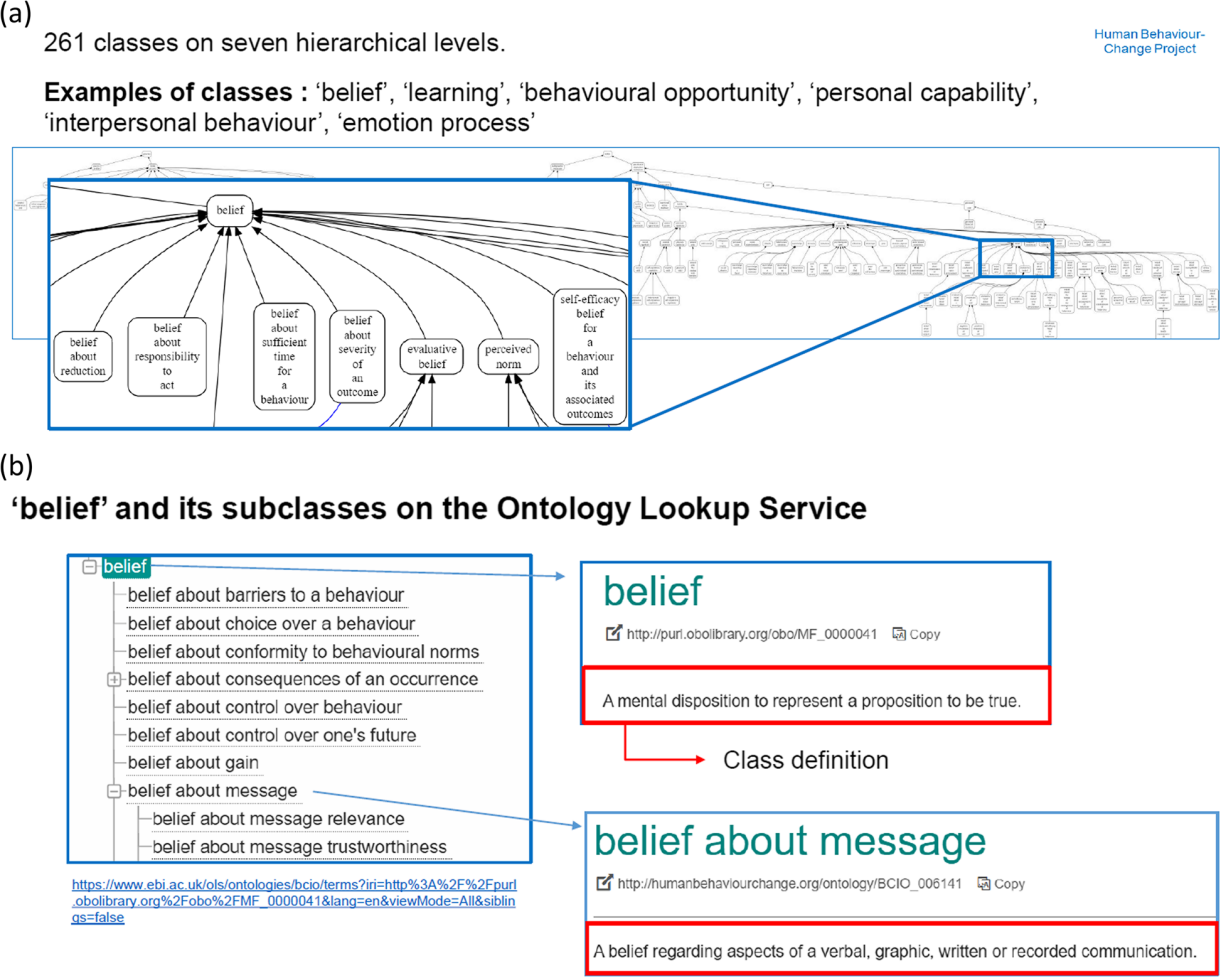
depicts part of the Behaviour Change Intervention Ontology, **a** showing the relationships between different classes and **b** showing the definitions of the two specific classes ‘belief’ and ‘belief about message’

Advantages of such ontologies are that they are unambiguous, computer-readable, and can easily be reused and updated [5]. They can thus be easily adapted to different contexts, such as different cultural contexts, different target groups, or different target behaviours [5]. Ontologies have revolutionized collaboration and research in the biological sciences. A prominent example is the Gene Ontology [4], which has been used to automatically annotate publications and to aggregate data. It allowed the field to progress faster, and reach new insights based on cumulative knowledge, which would not have been possible by manually reviewing literature. Ontologies may have similar advantages in the behavioural sciences. For that reason, a consensus report of the National Academy of Sciences has called for a strong and collaborative investment in ontologies [6]. As yet, there are not many ontologies in the behavioural sciences [7, 8]. A notable exception is the “Human Behaviour Change Project” (HBCP)^1^ [9], which aims to develop an ontology of behaviour change interventions (see Fig. 1).

Ontologies have the potential to accelerate health promotion research by (1) providing a controlled, unambiguous vocabulary, (2) automatizing annotation and aggregation of knowledge and (3) formalizing theories and findings.

First, ontologies can provide a controlled vocabulary for health promotion research. Without a controlled vocabulary, researchers may unintentionally assume that use of the same term reflects sameness or that use of different terms reflects differentness. Such jingle-jangle fallacies are abound in the health sciences. For example, ‘Stress’ can refer to physical pressure or tension, or a state of mental or emotional strain or tension. Although different terms, ‘Problem Solving’ and ‘Coping Planning’ refer both to identifying barriers and generating and selecting strategies to overcome them. Ontologies may counter such fallacies, and hence improve communication amongst researchers and practitioners, data integration and analyses, and the dissemination of knowledge. Importantly, a controlled vocabulary does not mean that a term cannot have multiple meanings. Instead, it allows us to specify which of the meanings we are referring to at any given moment. The National Academy of Science explicitly supports ‘ontological pluralism’, acknowledging that multiple, even competing, ways of understanding and representing reality exist [6]. In doing so, ontologies foster transparency about the used concepts, definitions and the decisions made by the developers. Therefore, documenting the background, scope and aim of ontologies, including cultural context, year of creation and updates, is an important task.

Second, ontologies can support the retrieval and the automatic aggregation of knowledge. An example of this is the HBCP, described above, which aims to automatically annotate publications concerning behavior change interventions using natural language processing [9]. The cumulative knowledge of the field being more easily and more clearly available, ontologies can then help inform, design and evaluate interventions [6].

Third, it is possible to formalize theories and findings by linking them to ontological classes and using ontological relationships to connect these. In a related effort, seventy-six behaviour change theories have been formally visualized and entered into a searchable database [10], allowing for systematic searches and easy comparison between theories.

Ontologies may also have specific advantages for physical activity (PA) research and interventions. An ontology on PA might have information on the activity itself, the person carrying out the activity, and the context of the activity. It can also link to other ontologies, for example of behaviour change interventions [11] or anatomy [12]*.* They can then be used to support the automatic detection and recognition of activities and their context, and improve the interoperability of sensor data (e.g. [13].)*.* This can be valuable for improving further innovations in the field (e.g. event-based ecological momentary assessment, and Just-in-Time-Adaptive-Interventions). Furthermore, ontologies may help in designing context-aware and personalized interventions. Context-aware and personalized interventions to promote PA are at their early stages, but have already been found to be more effective for both behavior [14, 15] and health outcomes [16] than their non-personalized counterparts. Ideally, the interaction between or combination of various factors should be considered, including sociodemographic and cultural factors, factors relating to physical and mental well-being and contextual factors. To achieve this, we need information on “who is active, what do they do, and under which circumstances?”. Unsurprisingly, the required knowledge is vast and complex. Ontologies offer a way to organize and structure such a complex network of information. and ontological reasoning may serve as a base for personalized recommendations.

While ontologies are a promising avenue to accelerate research in PA promotion, the field can be difficult to navigate. Repositories such as BioPortal [17] and the Open Biological and Biomedial Ontology (OBO) Foundry [18] have been established to easily find ontologies. OBO Foundry hosts ontologies that adhere to predefined criteria of best practice [18, 19]. High-quality ontologies are needed as they are meant to be adapted and maintained. If an ontology is of poor quality, researchers attempting to reuse the ontology will face difficulties. Outside of the OBO Foundry, it is difficult to identify ontologies that are both relevant and of high quality.

### Current review

In the current paper, we reviewed ontologies that are relevant to PA. The review has the following objectives: 1) to identify ontologies in the literature, 2) to assess their content and 3) to assess their quality, especially with regard to reusability.

## Method

The PRISMA reporting criteria for systematic reviews [20] were used, and the protocol is available via open science framework.^2^

### Identification of ontologies

The following electronic databases were searched in June 2021: CINAHL (via EBSCOhost), ProQuest Psychology, Web of Science, Scopus, PubMed, EMBASE, IEEE (Institute of Electrical and Electronics Engineers) Xplore, and ACM (Association for Computing Machinery).

The search strategy included strings related to ‘physical activity’ and ‘ontologies’. We excluded publications that contained the strings “gene” “dna” or “rna”. The final search strategy for Web of Science can be found in the appendix.

Results of the search were exported to EndNote and duplicates were removed. They were then imported into rayyan [21]. Screening was first based on the title and abstract only, and then on full texts. A second researcher screened 25% of the records *based on the title and abstract only*. The used ontologies were identified and indexed.

Two further methods were used:

- 1. Reference Searching: References from relevant records were screened for missing ontologies.
- 2. Key ontology repositories (i.e. OBO Foundry and BioPortal) were searched for terms related to ‘physical activity’.

#### Inclusion criteria

Publications were included if they met the following criteria:

- Original work that describes an ontology, using definitions of concepts and presenting relationships between concepts.
- The ontology describes PA or sedentary behavior, or has been used in a behaviour change intervention targeting PA or sedentary behavior.
- The ontology is available in English. The choice was made because of the ease of integrating and reusing these ontologies.

### Coding and quality assessment

Coding of ontologies occurred based on publicly available information. We coded *formal characteristics*, such as the ontology provider, country of origin, year of publication, version accessed and corresponding e-mail. Also, the number of classes and properties was coded*.* Concerning the content of the ontology, we differentiated between the physical activity domain, the profile domain, and three the context domain. The latter domain contained three subtypes (temporal, social and physical context). A description of these domains can be found in Table 1.

**Table 1:** Description of the different domains

Name Domain	Description	Examples
*Physical Activity Domain*	This domain describes different physical activities and their associated characteristics	Type of activity Intensity Duration
*Profile Domain*	This profile domain includes the aspects that are related to the individual (i.e. the actor of the physical activity), and their personal background. This includes amongst others psychological and physical characteristics. Any factor that describes an individual independently of its immediate context will be part of this domain. We thus decided to code content related to cultural or religious identity as profile factors, as well These factors can be either relatively stable, such as personality, or dynamic on a moment to moment basis, such as mood and fatigue	Health Attitudes Profession Socio-cultural background
*Context domains*		
*Temporal Context Domain*	The temporal domain describes concepts related to time and timing. Time does not have to be defined with standard temporal units such as hours or minutes but may also be defined using concepts in relation to other activities such as ‘after lunch’, or ‘when coming home’	Seasons Timestamps Working hours
*Physical* *Context Domain*	This domain describes the physical environment of an individual. This includes factors related to the built environment or natural environment, either stable factors, such as infrastructure, or dynamic factors, such as weather	Weather Location
*Social* *Context Domain*	The context domain describes the social context, either stable or dynamic factors	Friends Family Cohabitants

The coding of the quality of the ontologies was largely based upon the OBO Foundry principles of good ontology practice [22]. We included 12 of the 14 criteria. Principles were slightly adapted to be applicable to all ontologies. The principles “Relations” (Relations should be used from the Relations Ontology) and “Commitment to Collaboration” (Foundry ontologies are expected to collaborate with other Foundry ontologies) were considered not relevant for this review. After an initial quality assessment, the authors of the ontologies were contacted and provided with the opportunity to share additional materials or documentation.

1. Open: The ontology should be openly available on the internet. Being available upon request was not sufficient.
2. Common Formal Language: The ontology should be available in an owl file using the RDF-XML syntax.
3. Unique URI: This criterion was met if each class and property had a unique uniform resource identifier (URI), which is a unique characters sequence that distinguishes one resource from another.
4. Versioning: Versions should be labelled clearly, including their date of publication and the changes made.
5. Textual Definitions: An ontology should have definitions for the majority of its classes, in particular for top level terms.
6. Naming Conventions: An ontology should have clear naming conventions. This criterion was met if names were unique and intelligible to the coding team MB and SC.
7. Documentation: Significant documentation should be available, e.g. in a published paper describing the ontology, websites, or in manuals for developers and users.
8. Locus of Authority: We coded whether contact details (at least a name and email address) of a person were provided. A corresponding author of a publication was sufficient, but the email address needed to be valid (i.e. not return an error notification).
9. Reuse: We coded whether ontology developers reused ontological or non-ontological resources during development. This criterion was met if there was clear documentation that content was imported from other ontological or non-ontological resources.
10. Documented Plurality of Users: We coded whether the usage of the ontology by multiple independent people or organizations was documented in a freely available online document. This information had to be provided by the ontology developers, not those using the ontology.
11. Maintenance: Ontology providers should have a plan for maintaining the ontology. and provide this information in the documentation. We also coded whether maintenance did take place, e.g. regularly update.
12. Responsiveness: We coded whether ontology developers offered channels for community participation and were responsive to requests. This criterion was met if developers had set up a way to track community requests and suggestions (e.g., issue tracker).

After the initial coding of the quality assessment, the authors of the ontologies were contacted with the coding results of their ontology, and provided the opportunity to share additional materials or documentation. After reviewing these responses, the results were finalized.

## Results

**Fig. 2:**
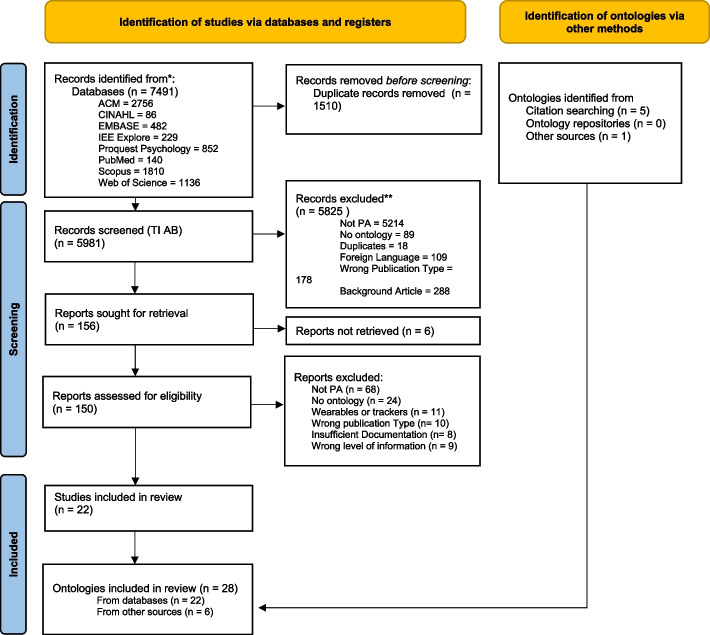
PRISMA Chart

Figure 2 displays the number of publications identified, screened and excluded at each stage of the review process, as well as the number of ontologies identified through each method and in total.^3^

### Ontologies related to physical activity

We identified and assessed the quality of 28 ontologies. A brief summary of each ontology can be found in Table 2. A brief description of each ontology can be found in Additional File 2.

**Table 2:** Overview of identified ontologies

Name	Reference	Context of Develop-ment	*No. of classes*	*No. of properties*	Country of Origin	Year of publication
Exercise and food ontology	[23]	Knowledge Exchange/ Information Retrieval	-	-	Poland	2020
FASTO	[24]	Personalized Recommendations (Clinical)	9577	658	South Korea	2019
PACO	[25]	Knowledge Exchange/ Information Retrieval	224	23	US	2019
OPTimal	[26]	Personalized Recommendations (Clinical)	142	10	Greece	2019
OMDP	C	Knowledge Exchange/ Information Retrieval	-	-	China	2019
Alian	[27]	Personalized Recommendations (Clinical)	-	-	USA	2018
OAFE	[28]	Personalized Recommendations (Activity Promotion)	405	61	France	2018
exercise ontology	[29]	Knowledge Exchange/ Information Retrieval	-	-	India	2018
ECOPPA	[30]	Personalized Recommendations (Activity Promotion)	-	-	Canada	2018
Sloth	[31]	Personalized Recommendations (Activity Promotion)	-	-	Germany	2017
Mining Minds Context Ontology	[32, 33]	Knowledge Exchange/ Information Retrieval	225	25	South Korea	2017
Ontology of Motivational Messages	[34]	Personalized Recommendations (Activity Promotion)	-	-	Spain	2017
Patient Domain Ontology	[35]	Knowledge Exchange/ Information Retrieval	-	-	China	2017
TrhOnt	[36]	Personalized Recommendations (Clinical)	2351	65	Spain	2016
Health and Meal Ontology	[37]	Personalized Recommendations (Activity Promotion)	-	-	Mexico	2016
Exercise Search Ontology	[38]	Knowledge Exchange/ Information Retrieval	-	-	Germany	2015
SMASH	[39]	Knowledge Exchange/ Information Retrieval	189	144	USA	2015
SHADE	[40, 41]	Personalized Recommendations (Clinical)	-	-	Pakistan	2014
SHCOntology	[42]	Wearables and sensors	-	-	Spain	2014
UFIT	[43, 44]	Personalized Recommendations (Activity Promotion)	-	-	Taiwan	2014
TRAK	[45–47]	Knowledge Exchange/ Information Retrieval	1619	16	UK	2013
OPA Ontology	[48]	Knowledge Exchange/ Information Retrieval	-	-	Portugal	2013
OPE	[49]	Knowledge Exchange/ Information Retrieval	634	27	USA	2013
Healthcare Common Ontology	[50]	Wearables and sensors	13	-	South Korea	2011
Exercise Plan Ontology	[51]	Personalized Recommendations (Clinical)	-	-	Greece	2011
ActivO	[13]	Wearables and Sensors	-	-	Italy	2011
Nuadu Ontology Collection	[52]	Knowledge Exchange/ Information Retrieval	-	-	Finland	2007
Ontology for health and exercise	[53]	Personalized Recommendations (Activity Promotion)	-	-	Japan	2006

### Content domains covered in the ontologies

#### Physical activity domain

All ontologies distinguished between types of activities; 18 between type of physical activities (e.g. cycling, running, swimming), 10 between types of exercises (e.g. bicep curls, squats) and 7 between all types of activities, whether physical or not (e.g. eating, sleeping). Also well integrated was the intensity of the activity (*n* = 14). Less covered features were the effects or function of an exercise (e.g. increased heart rate, stretch muscle, flexibility improvement, *n* = 5), the type of exercise (e.g. stretching, strengthening, *n* = 6), equipment needed (*n* = 5), associated parts of the musculoskeletal system (*n* = 6), function of activities (e.g. occupational activity, transport, *n* = 5), associations with specific workouts (*n* = 3), phases or sessions (*n* = 1), kinds of movements performed in the exercise (e.g. flexing, *n* = 3), contraindications (*n* = 2), required user experience (*n* = 1), and linked animations/visualisations (*n* = 1).

#### Profile domain

Nineteen ontologies contained profile information. Basic sociodemographic information (*n* = 18) was most often included, such as age (*n* = 15), sex or gender (*n* = 14), administrative identifiers, such as patient or client IDs or national register numbers (*n* = 9), occupation (*n* = 7), education (*n* = 3), cultural or religious background (*n* = 3), ethnicity (n = 2), address (*n* = 2), household members (*n* = 1), marital status (*n* = 1), socioeconomic status (*n* = 1).

Ontologies also often contained some form of clinical or health information (*n* = 21). More than half of the identified ontologies included current diagnoses (*n* = 15). Others included health-related risk factors for specific diseases (*n* = 7). Many included health characteristics (*n* = 16), including body mass (*n* = 13), blood pressure or blood glucose levels (*n* = 10), height (*n* = 9), body fat (*n* = 3), and fitness level (*n* = 3) of the user. Some ontologies considered current treatments (*n* = 4) and recommendations by health care providers (*n* = 3).

Psychosocial features were not often presented in the ontologies (*n* = 5). Some of those were general, such as emotional and psychological state (*n* = 2) or feelings of insecurity (*n* = 1). Others included specific information related to physical activities, such as determinants for PA (e.g. motivation, intention, self-efficacy, *n* = 2), fear of falling (*n* = 1) or fear of fatigue (*n* = 1). Six ontologies included preferences for specific activities or intensities, five included goals concerning PA or health outcomes (e.g. weight loss), and six included current lifestyle and habits.

#### Context domains

The physical context was integrated in 12 out of 28 ontologies. This was usually done by specifying the location, e.g. a building or place from a list (*n* = 8), specifying whether it was indoors or outdoors (*n* = 8) or using GPS location (*n* = 2). Weather was also often included, including weather in general (*n* = 4), lighting (*n* = 3), and temperature (*n* = 4). The surface features (e.g. water, ice, snow) and type of soil needed were each covered in one ontology. Finally, one ontology included the usability, accessibility and safety of a given location (47).

The temporal context was covered by 14 out of 26 ontologies. Most of the ontologies covered basic temporal aspects such as duration of an activity (*n* = 8), start (*n* = 5) and end time (*n* = 2) or the frequency or regularity of an activity (*n* = 4). Four ontologies defined the day that the activity took place on. Events, seasons, or times of the day were each covered by one ontology.

Five ontologies covered the domain of social context. Family, social purpose of an activity and social interactions were included in two ontologies. Social support, social networks, communities, cohabitants, groups and social events were each covered by one ontology.

### Quality assessment of ontologies

The assessed quality of the ontologies are summarized in Table 3, including the total number of criteria met per ontology, and the total number of ontologies meeting each criterion.

**Table 3:** Quality assessment of the identified ontologies

Name	**Reference**	**Total criteria met**	**Available**	**Format**	**URI**	**Versioning**	**Reuse**	**definitions**	**Documentation**	**PluralityUsers**	**LocusOfAuthority**	**Naming**	**Maintenance**	**Responsiveness**
Exercise and food ontology	[23]	3							x		x	x		
FASTO	[24]	8	x	x	x	x	x		x		x	x		
PACO	[25]	9	x	x	x	x	x		x		x	x	x	
OPTimal	[26]	7	x	x	x	x			x		x	x		
OMDP	[54]	4					x		x		x	x		
Alian	[27]	3					x		x		x			
OAFE	[28]	5					x	X	x		x	x		
exercise ontology	[29]	4					x		x		x	x		
ECOPPA	[30]	3							x		x	x		
Sloth	[31]	0												
Mining Minds Context Ontology	[32, 33]	6	x	x	x				x		x	x		
Ontology of Motivational Messages	[34]	2							x			x		
Patient Domain Ontology	[35]	3							x		x	x		
TrhOnt	[36]	8	x	x	x		x	X	x		x		x	
Health and Meal Ontology	[37]	3							x		x	x		
Exercise Search Ontology	[38]	3					x		x		x			
SMASH	[39]	9	x	x	x	x	x		x	x	x	x		
SHADE	[40, 41]	3							x		x	x		
SHCOntology	[42]	3							x		x	x		
UFIT	[43, 44]	4					x		x		x	x		
TRAK	[45–47]	9	x	x	x	x	x	X	x		x	x		
OPA Ontology	[48]	3					x		x			x		
OPE	[49]	6	x	x	x	x	x					x		
Healthcare Common Ontology	[50]	3							x		x	x		
Exercise Plan Ontology	[51]	2							x			x		
ActivO	[13]	3							x		x	x		
Nuadu Ontology Collection	[52]	2							x		x			
Ontology for health and exercise	[53]	2							x			x		
Total (out of 28)			8	8	8	6	13	3	26	1	22	23	2	0

There was strong variability in the extent to which quality criteria were met. Notably, only eight out of 28 ontologies were freely available online. The lack of information strongly affected the assessment of the remaining criteria. This influenced specifically the rating on the criteria for common format, URI, versioning and clear definitions. Ontologies met an average of 4.23 (SD = 2.47) and median of 3 (Q1 = 3, Q3 = 6) out of 12 criteria, with a minimum of 0 and a maximum of 9 out of 12 criteria. The criteria met by most ontologies are documentation (*n* = 26), clear naming (*n* = 23) and locus of authority (*n* = 22). The least met criteria were responsiveness (*n* = 0), maintenance (*n* = 2) and providing clear definitions (*n* = 3). Ontologies meeting most criteria are the Physical ACtivity Ontology” (PACO), “Semantic Mining of Activity, Social, and Health data” (SMASH) ontology [39] and the “Taxonomy for Rehabilitation of Knee conditions” (TRAK) [45–47] ontology, meeting nine criteria each. The lowest number of criteria was met by the Sloth ontology [31] which meets none of the quality criteria.

## Discussion

Ontologies have the potential to increase the efficiency of research in the field of PA. However, few PA researchers are familiar with ontologies, and the field can be difficult to navigate. In the current paper, we identified relevant ontologies in the field of PA, assessed their content, and rated their quality. We identified 28 ontologies. There was a substantial variability in scope and content of the identified ontologies, ranging from knowledge systems that formally represent knowledge about a specific disease, and can reason using the knowledge residing in that ontology [54] to ontologies specifically created to describe physical activities [8, 49] or detect behavior in a particular context [13]. There were also differences in the content covered by the ontologies. All ontologies included the activity domain, albeit in varying detail, and most covered some user profile information. Context information was covered by fewer ontologies and in less detail. No single ontology comprehensively captured PA in the context where it occurs, including physical, temporal and social aspects. Such variability was expected, as most ontologies were created for specific use cases that did not require all information. Because ontologies can be integrated and connected, it is not necessary for each ontology to contain all information relevant for PA. However, it should be avoided that identical or very similar concepts are defined independently from each other and without referencing to each other. By importing concepts from established existing ontologies, ontology developers can improve the interoperability and clarity of the ontology. For example, when different ontologies are developed for each context domain, they can easily be integrated if they all refer to the same definition of PA.

We have found that while many ontologies meet the criteria that enable them to function in its original system, such as providing the ontology in a common format and using unique identifiers, many criteria relevant for reuse, are not met. Neither do most researchers seem to maintain their ontologies. Given that the goal of ontologies is to provide unambiguous concepts, ensure reusability and reduce redundant research [55], it is surprising that many ontologies did not meet these criteria.

Remarkably, 20 out of the 28 ontologies were not published in repositories such as OBO Foundry or BioPortal. These ontologies were also not freely available elsewhere, such as on GitHub or project-specific websites. This limited our ability to adequately assess the quality criteria. Most likely these ontologies were not designed to be shared with other users. In line with this view, some authors noted in their documentation or email communication with us that their ontologies were created as a proof of concept, or to demonstrate the interaction with a specific system. They were not designed to provide a comprehensive ontology of a particular phenomenon. We, hence, strongly recommend to consider ontologies available via an ontology repository over those described in a paper but not made available.

### Description of the three highest scoring ontologies

Notwithstanding that none of the ontologies met all our criteria, there were some good scoring ontologies that may serve as a good starting point for further development in the field of PA. The three best scoring ontologies are the PACO [25], SMASH ontology [39], and the TRAK ontology [45].

The “Physical ACtivity Ontology” (PACO) [25] was created to structure and standardize descriptions of PA. It extracted concepts from existing PA questionnaires and scales using natural language processing. It contains an extensive list of physical activities, including daily living activities that require the actor to be physically active. It also contains the effect of exercise, equipment, and program, and provides information about the amount, frequency, regularity, intensity, required condition (snow, ice, water, ground) and location (inside or outside) of an activity. The authors demonstrated a use case where PACO successfully standardized and classified PA descriptions.

The “Semantic Mining of Activity, Social, and Health data” (SMASH) ontology [39] was created for human behavior prediction. The ontology includes social and physical activities. SMASH is an ontology for health social networks, containing three modules, namely biomarkers, social activities and physical activities. Specifically, it contains lists of exercises, physical activities and daily living activities, sociodemographic information about the individual, social activities such as social events, interactions and relationships, and social entities such as people or communities. SMASH improves the prediction and explanation of behavior and interventions in the context of sustained weight loss.

The “Taxonomy for Rehabilitation of Knee conditions” (TRAK) ontology [45] aims to provide a framework that can be used to improve efficiency in research by collecting coded data. TRAK was developed following the OBO Foundry design principles and was informed by experts. It contains a list of events relevant for PA, such as accidents or forceful joint movements, an extensive list of exercises and sports, lists of joint movements and muscle contractions as well as anatomical entities. TRAK also contains information such as the roles of healthcare providers, and healthcare activities. It has been developed further into the KneeTex ontology [56], but these changes were out of scope for this review. TRAK has been integrated into a web-based intervention that provides patients with health information, personalized exercise plans and remote clinical support [46].

The three ontologies vary in scope and context of development. It should be noted that even within the three highest scoring ontologies, only one formulated a plan for maintenance [25], contained definitions for the majority of its classes [45], and documented the different independent users [39] respectively. None met the criterion of responsiveness. Likely, not all criteria are equally important, and their importance may depend on the goal of the research. For example, some criteria, namely using a common format and unique URIs, are necessary in order for the ontology to function in its original context. However, other criteria are critical if ontologies are meant for reuse, such as the availability of the ontology, and classes with clear naming conventions. Criteria relevant for transparency are more related to having clear documentation of the development and evaluation of the ontology available, providing clear version control, providing clear definitions for classes and having a locus of authority that researchers can reach out to if they have questions. Lastly, having and following a clear maintenance plan and being responsive to user requests are criteria that relate to improving the ontology and keeping it up to date with current scientific standards. While meeting these criteria has the potential to vastly improve an ontology, it is also associated with significant efforts.

### Implications for further research

Ontologies have strong potential for PA promotion. Some benefits are generic. Ontologies provide clear definitions of concepts, allow simple and complex relationships between concepts, facilitate the aggregation of knowledge and represent the knowledge in a computer-readable format for future (re)use and adaptation. This has been done successfully in other disciplines, such as the field of genetics [3, 4], allowing for progress by facilitating evidence synthesis and aggregating existing knowledge. Within health behavior change, the HBCP [9] is the first to make use of ontologies. The goal of HBCP is to provide answers regarding which interventions work for whom under which circumstances. Within that project, knowledge is structured into behaviour, mechanisms, intervention, exposure and context domains [9]. Multiple ontologies have already been developed in this project [11, 57–59].

Use of ontologies in the field of PA may be relevant for the following reasons: ontologies can support (1) automatic recognition of PA and its context and (2) intervention development and clinical practice. First, automatically detecting and recognizing PA can be valuable for both research and applied settings [60], and is a promising avenue [61]. By having data via devices on the amount and intensity, as well as the type of PA, researchers and clinicians do not have to rely on self-report, which can be skewed due to different factors such as memory bias. It can therefore give a less obtrusive and more complete picture of the PA of an individual. Ontologies have the potential to improve automatic activity recognition [13]. Second, ontologies can support (digital) PA promotion. Because ontologies are computer-readable, they can easily be integrated into systems that provide decision support, and have been already developed in a research context (e.g. [33, 40]). Ontologies can help to provide recommendations for specific exercise plans (e.g. [41]) for particular patient groups, or may help to plan PA more generally.

In this review, we found no ontology or set of ontologies that covered all important aspects of PA. There is a clear need for a set of ontologies that fully captures PA in its context, including the physical, temporal and social domain. Such an ontology can integrate information that is covered by the higher quality ontologies identified in this review, and build upon those following the quality principles described above.

This review can serve as a first introduction to ontologies for PA researchers, and specifically those focusing on PA promotion. We have provided an overview of existing ontologies on PA, their content and their quality. To facilitate systematic identification of useful ontologies in the future, we recommend researchers to start using an ontology repository. We recommend starting with OBO Foundry [22] and then searching BioPortal [17], as OBO Foundry guarantees for the quality of their ontologies [19]. Since the current review has been conducted, another foundry has launched, specifically targeting behavoural and social sciences, namely the Behaviour and Social Sciences Ontology (BSSO) Foundry.^4^ While it does not yet contain many ontologies, it should also be considered when searching for suitable ontologies for re-use. In a second step, researchers should determine whether the content of the ontology meets their needs by investigating its respective classes. If the ontology is deemed relevant, researchers need to evaluate whether ontologies are of sufficient quality for reuse. Ontologies published in a repository are usually available online in a common format and contain URIs, as those criteria need to be met for an ontology to function. In order to be suited for reuse, ontologies should at least contain clear names, and documentation of the development, structure and evaluation of the ontology should be available to the researchers. Meeting other quality criteria defined in this review, especially containing clear definitions and following a transparent maintenance plan, provide strong additional value. However, because only few ontologies meet these criteria, researchers might not necessarily want to exclude all ontologies that do not meet them. In that case, expanding or updating the ontologies might be necessary before implementing them. We encourage collaboration with ontology engineers or other researchers with expertise in ontology development whenever changes need to be made to an ontology, or if new ontologies need to be created. Lastly, we strongly recommend to use the OBO Foundry principles for development, adaptation and evaluation of ontologies. Most importantly, we encourage researchers to make their ontology and relevant documentation freely available online, preferably on an ontology repository.

## Strengths and limitations

This is one of the first systematic reviews assessing the content, methods and quality of ontologies within behavioral sciences. Due to the novelty of the topic, there are few guidelines on how to best review ontologies. We, therefore, decided to perform a broad search, including ontologies identified from research articles, ontology repositories and citation searching. We also decided to include ontologies even if they were not publicly available. This allowed us to draw a comprehensive picture of existing ontologies in the field of PA, and to discover some pitfalls not identified earlier [8]. However, due to the nature of a review, we could only assess content, methods and quality based on the documentation that was publicly available. Because the amount of documentation strongly varied, we might have coded well-documented ontologies as disproportionately more qualitative in comparison to less well-documented ontologies. While we tried to compensate for this by contacting authors and asking for additional information, the response to our requests were unfortunately low. Lastly, this review only included English language ontologies. We may have missed ontologies in other languages. not English language that it adequately covers non-English language literature.

## Conclusions

We identified 28 ontologies on PA and assessed their content, methods and quality according to twelve defined quality criteria. We found that most ontologies cover the activity and profile domains of PA, whereas the context domains are covered by less ontologies and in less detail. No ontology covers all domains of PA extensively enough to paint a comprehensive picture of physical activities. Whereas most ontologies meet technical criteria for quality, many fail to ensure transparency and reusability, with only eight ontologies being publicly available.

Recommendations for researchers in the field of PA include steps to identify and evaluate ontologies. We also encourage to collaborate with ontology engineers if ontologies need to be adapted, updated or created. Finally, we call for researchers to make their ontologies and extensive documentation freely available online whenever possible in order to facilitate reuse and adaptation of their ontology.

## Supplementary Information

**Additional file 1.** 

**Additional file 2.** 

## Data Availability

The protocol and coding scheme used in this study areavailable in the osf repository, [https://osf.io/6ej38/].

## Abbreviations

HBCP: Human Behaviour Change Project
OBO: Open Biological and Biomedical Ontology
PA: Physical Activity
PACO: Physical ACtivity Ontology
SMASH: Semantic Mining of Activity, Social and Health Data
TRAK: Taxonomy for Rehabilitation of Knee conditions
URI: Uniform Resource Identifier

## Footnotes

1 https://www.humanbehaviourchange.org/

2 https://osf.io/ztp9e

3 Ontologies identified via other sources were identified by one of the authors during screening.

4 http://www.bssofoundry.org/
