# Supplementary material for: Content and quality of physical activity ontologies: a systematic review
Source: Int J Behav Nutr Phys Act. 2023 Mar 13;20:28. doi: 10.1186/s12966-023-01428-y (PMC10009987; doi:10.1186/s12966-023-01428-y)
Supplement: Supplementary file 1 — Additional file 1. [file 12966_2023_1428_MOESM1_ESM.pdf]

Search Strategy Web Of Science

(TS="physical* near/3 ACTIV*" OR TS="leisure activities" OR TS="exerc*" OR TS="walk*" OR TS="bicycling" OR TS="commut*" OR TS="active transportation" OR TS="active travel" OR TS="sport*" OR TS="fitness" OR TS="exertion" OR TS="functional movement" OR TS="sedentary" OR TS="motOR TS=activit*" OR TS="physical condition*" OR TS="physical endurance" OR TS="movement therap*")

AND (TS=ontolog*)

NOT (TS="gene" OR TS="dna" OR TS="rna")
