# Supplementary material for: Content and quality of physical activity ontologies: a systematic review
Source: Int J Behav Nutr Phys Act. 2023 Mar 13;20:28. doi: 10.1186/s12966-023-01428-y (PMC10009987; doi:10.1186/s12966-023-01428-y)
Supplement: Supplementary file 2 — Additional file 2. [file 12966_2023_1428_MOESM2_ESM.pdf]

Additional File 1. Structured Overview of all ontologies

Ontology Alian (62)

Aim: This paper proposes a proactive diabetes self-care recommendation system specifically for American Indian patients. The system creates personalized recommendations for diet and physical activity.

Content and Domains: This ontology incorporates the profile and activity domains. For the user, it includes general demographic information, existing diagnoses and physical characteristics such as bodymass and height, and exercise preferences. For the activities, this ontology provides a list of different physical activities and categorizes activity into the domains occupational, athletic and exercise activity.

Development: For ontology development, a multi-phased iterative and incremental ontology design and development methodology was used, going through the following six work phases: (1) scope definition, (2) knowledge acquisition, (3) specification, (4) conceptualization, (5) implementation, and (6) evaluation. Different personnel may get involved in different phases. Some existing ontologies about food, nutrition and workouts for re-used.

Implementation and Validation: The system was implemented as a mobile application and evaluated using case studies and human expert verification.

Telerehabilitation Ontology (45)

Aim: This ontology aims to create an overview of knowledge in the field of physiotherapy. This system can then be used for clinical decision support systems

Domains& Content: This ontology incorporates the activity and profile domain. It contains general patient information, such as gender and occupation, but also medical information such as diagnoses. It also includes objectives. TrhOnt includes information on different exercises, including exercise types, musculo-skeletal parts associated with the exercise, its intensity and the movements that are carried out as well as a workout that the exercise can be part of.

Development: For development, it followed the NeOn methodology, integrating knowledge from existing ontologies and non-ontological resources, as well as additional physiotherapy-related knowledge.

Implementation and Validation: In order to validate the ontology, four use case scenarios were developed and competency questions were formulated. It was further evaluated using OOPS!, and quality criteria were evaluated (including accuracy, adaptability, clarity, completeness, computational efficiency, conciseness, consistency).

Sloth – The Interactive Workout Planner (46)

Aim: Sloth is an interactive workout planner that is based on an ontology. In SLOTH's application scenario, the user wants to select a set of exercises fort he current training session in order to reach their fitness objective.

Domains& Content: This ontology focuses on the activity domain. It incorporates specific kinds of exercises and which part of the musculo-skeletal system they engage. It also defines different workouts and maps which exercises can be a part of which workouts. The system also gives the user information concerning why certain exercises are suitable for certain goals. The SLOTH system also integrates the temporal domain by referring to the date of specific trainings.

Development: The development or validation of SLOTH are not further described in the paper at hand.

Implementation and Validation: The development or validation of SLOTH are not further described in the paper at hand.

Taxonomy for RehAbilitation of Knee conditions (44,58,77)

Aim: TRAK (Taxonomy for RehAbilitation of Knee conditions) can be used to support knowledge exchange between experts (healthcare professionals and researchers) as well as support structured training in physiotherapy of knee conditions. It can also be used to mine text from other relevant sources. TRAKs main use is to support epidemiologic studies. It is openly available via its own website and bioportal.

Domains& Content: TRAK incorporates the activity domain. It formally models information relevant for the rehabilitation of knee conditions. It contains a list of activities and exercises, as well as the movements carried out in the context of these activities and exercises and a further classification (e.g. agility, stretching, weight resistance). TRAK also integrates illustrative images of specific types of concepts.

Development: TRAK follows the design principles recommended by the Open Biomedical Ontologies (OBO) Foundry, using the BFO as a foundation and integrating relevant ontologies such as Information Artifact Ontology; Ontology for General Medical Science and Phenotype And Trait Ontology. The authors conducted a systematic literature review and integrated input by important stakeholders such as clinicians.

Implementation and Validation: For validation, TRAK was successfully piloted in a physiotherapy service for registering treatment modalities.

.

OAFE: An Ontology for the description of elderly activities (63)

Aim: OAFE has been designed to model the activity domain of older adults in order to adapt the suggestion of activity according tot he state of health, the level of activity and autonomy, the environment and het preferences of the elderly person.

Domains& Content: OAFE covers all domains described in this review. For the profile domain, demographic data (e.g. age and sex), physical characteristics (e.g. bodymass, height, blood pressure), but also objectives and preferences are described. OAFE also describes some psychological factors specific to older adults such as a fear of falling. OAFE describes activities based on a list of physical and not-physical activities, and also further describes them using intensity and domains. For the location domain, a distinction is made between inside and outside as well as other context factors such as the temperature and type of soil. For the social domain, the network and cohabitants of the individual can be mapped. Lastly, for the temporal domain, OAFE can describe the frequency of certain activities.

Development: For development, the NeOn methodology was followed, making use of use case scenarios and competency questions. Some concept from the SMASH ontology, the PROV-O vocabulary as well as the CPA resource have been selected for re-use in the design of OAFE.

Implementation and Validation: To evaluate OAFE, a series of queries were made to respond to competency questions.

Exercise ontology (64)

Aim: The aim of the ontology is to support analysis of implicit constituents in exercise instructions.

Domains& Content: This ontology covers the activity domain. The exercise ontology describes specific physical exercises in terms of the body parts and joints that are used, the posture and movements, the start end position and the intensity. It also categorizes each activity as an aerobic, strengthening or stretching activity. It uses the temporal domain to describe the duration of a given activity.

Development: The exercise ontology used competency questions for development.

Implementation and Validation: The ontology was integrated into an application system for textual instruction to virtual action conversion.

FHIR and SSN-based T1D Ontology (60)

Aim: The FASTO ontology was built in the context of developing a mobile health application for type 1 diabetes monitoring and treatment, connecting patients to different service providers and different sources of medical data. Together with an ontology reasoner, FASTO can provide real-time knowledge-as-service to patients and physicians.

Domains& Content: FASTO incorporates the profile and activity domain. For the profile, FASTO includes general information such as identifiers, address, age and gender. It also includes clinical information such as current treatment, recommendations by healthcare providers, diagnoses and disease risk factors. For activities, FASTO contains a list of physical activities and exercises. The FASTO ontology extends the knowledge of existing ontologies focused on diabetes (DDO and DMTO) using a four-tier smart mobile and context-aware architecture to support rapid prototyping of m-Health applications in different scenarios.

Development: The authors documented that they followed design principles by the OBO Foundry.

Implementation and Validation: The ontology was evaluated using the OOPS! Reasoner and protégé metric. It was also evaluated by domain experts in medical practice as well as ontology engineering.

Semantic HealthCare Assistant for Diet and Exercise (72,73)

Aim: Semantic HealthCare Assistant for Diet and Exercise (SHADE) is developed in order to create helpful lifestyle suggestions for Type 1 diabetes patients. It can create food- and exercise recommendations.

Domains& Content: The SHADE ontology covers the profile, activity and location domain. It consists out of multiple domain ontologies, including the personal health profile, food, disease, and exercise domain. For the profile domain, SHADE contains general information such as identifiers, age and gender. It also contains existing diagnoses, information on the current lifestyle of the user and physical characteristics such as bodymass and height. For the activity domain, SHADE contains a list of activities and describes them further in terms of intensity and domain. Lastly, SHADE contains a (limited) list of possible locations and whether they are situated indoors or outdoors to map the physical context of a given activity.

Development: No information was available concerning the development of the ontology.

Implementation and Validation: The authors have successfully implemented the ontology into a prototype, and have validated the recommendations regarding nutrition, but not exercise. Authors have planned further validation and improvements with patients, endocrinologists and nutritionists.While parts of the ontology were evaluated, those parts were not of relevance for this review.

Simple Health Care Ontology (74)

Aim: The aim of the SHCOntology is to determine whether a person's heart rate puts them at risk during physical exercise.

Domains& Content: The SHCOntology covers the profile and activity domains. For the profile domain, it covers general information such as identifiers, age and gender. It also contains information on existing diagnoses and physical characteristics such weight and height. Concerning the activity, it maps the intensity and recommended heart rate for each one in order to determine whether the person's heart rate puts them at risk.

Development: Expert rules were created in order to determine intensity levels for physical activity. The ontology was demonstrated on two examples.

Implementation and Validation: The authors are planning to implement the full system in order to validate the ontology.

Ontology for health and exercise (82)

Aim: This ontology is part of a health advice system.

Domains& Content: This ontology covers the profile and activity domains. It maps various concepts and their relationships about health and exercise. The ontology consists out of six main concepts: a Person, Health Data, Exercise, EffectOfExercise, Goal and HealthAdvice. For the profile domain, it describes a general profile (not further described in documentation), existing diagnoses and objectives. On the activity domain, it contains a list of exercises and their effects.

Development: The development and validation of the ontology are not further described.

Implementation and Validation: The ontology has successfully been implemented.

Physical ACtivity Ontology (55)

Aim: The PACO ontology was developed in order to support structuring and standardizing heterogenous descriptions of physical activities.

Domains& Content: The ontology covers the activity, location and time domains. PACO describes exercises using 5 main concepts, namely Activity, Exercise_effect, Exercise_equipment, Exercise_program and Modifier.it contains an extensive list of physically active activities. For the context of the activity, it describes whether an activity can take place indoors or outdoors and whether there are required conditions such as ice or snow. For the time domain, PACO covers the regularity and frequency of an activity.

Development: The ontology was developed by using natural language processing tools on 1140 unique sentences collected from physical activity questionnaires and scales as well as existing terminologies and ontologies.

Implementation and Validation: PACO was evaluated for logical and structural consistency and best practices of building an ontology, as well as by applying it to a use case and evaluating its functioning.

Healthcare Common Ontology (79)

Aim: The healthcare common ontology is part of a healthcare context information model that is created to implement a ubiquitous environment. Within this u-healthcare service, patients (among other things) can receive personalized food and exercise recommendations. Other ontologies, namely the device ontology for the smartphone and the spatial ontology for the home, were also used within this system.

Domains& Content: The Healthcare Common Ontology covers the profile, activity, location and temporal domain. It describes eight main classes, namely, context, environment, location, device, activity, medicine, auxiliary and individual. For the profile, this ontology contains general sociodemographic information such as age, sex and occupation, but also clinical diagnoses and risks, and physical characteristics such as height, weight and blood pressure. Lastly, it contains information on the lifestyle or habits of the user. For the activity, a general list of activities – not exclusively physical activities – is included. The physical context is described in terms of specific locations, indoors and outdoors, temperature, weather and lighting. Lastly, time is described in terms of the season, day and start time of the activity.

Development: The development is not further described in the available documentation.

Implementation and Validation: The recommendations given by the system were validated.

Exercise Plan Ontology (80)

Aim: The present ontology is part of a framework designed to facilitate healthcare professionals in personalized exercise prescription. It creates exercise plan suggestions based on patient's profiles and supports readjustments of a prescribed plan based on the patient's response with respect to goal achievement and changes in physical medical status.

Domains& Content: The Exercise Plan Ontology contains information on the profile, activity, location and time domain. The profile domain describes sociodemographic factors such as age and sex, clinical diagnoses and risks, physical characteristics such as height, weight and blood pressure. Lastly, preferences and objectives are described. Activities are described in terms of the activity type and intensity, but also in terms of which workouts and sessions they belong to. The context of the activity is described in terms of their location (indoor/ outdoor) and their timing (frequency and duration). The major classes defined within the ontology are patient, exerciseplan_concept, medicalprofile_concept and auxiliary classes (such as time, range, goal).

Development: The development is not further described in the available documentation.

Implementation and Validation: The system is demonstrated on a use case. The ontology was approved by medical experts in early validation phases but needs to be further validated.

Exercise Search Ontology (71)

Aim: This ontology was created as part of an exploratory search engine that simultanously accesses internet resources and a local knowledge base (the ontology).

Content: The domain ontology contains detailed information on the activity domain. It categorizes exercises including textual descriptions of preparation and execution. The exercises are linked to required equipment, target and synergist muscle groups and an animation. The list used in the ontology is based on the exercises published at ExRx.net. Second component of the ontology is the user specific ontology which only contains exercises which have been added to the system during the search process. The third component is the DBpedia ontology, using a subset from the seeds physical exercise and physical therapy.

Development: There is no information on the development of the ontology in the available documentation.

Implementation and Validation: The system is demonstrated on an example from the fitness domain where the user searches for physical training exercises on the web to complete a personal training plan.

OPTimal (61)

Aim: The OPTImAL ontology focuses on the adherence of cardiovascular patients to physical activity and exercise training. It can be used as part of a clinical decision support in the medical practice of experts targeting CVD patients' adherence with physical activity regimen.

Content: OPTImAL covers the profile, activity, physical context and social domains. It consists of the top-level classes patient factor, studied population, factor relation type, activity adherence type, adherence quality, adherence level, activity behaviour, activity behavior dimesion, activity behavior stage and CRP duration.

The profile domain is covered in depth. It includes general profile factors such as age, sex, education, occupation, ethnicity, marital status, socio-economic status and cultural background, as well as physical characteristics such as height, weight and blood pressure. It also contains clinical information such as current treatment and recommendations by healthcare providers and clinical diagnoses, as well as information on the current lifestyle and psychological characteristics. The activity domain consists of a list of activities during and after cardiac rehabilitation. The location domain contains a list of possible locations for an activity, and the social domain contains information on the family and social support of the individual.

Development: OPTImAL was developed using the NeOn framework.

Implementation and Validation: The ontology was validated by an independent cardiologist and three CR trainers for appropriateness and usefulness, and was also validated for consistency.

Exercise and food ontology (59)

Aim: These two domain ontologies were developed as part of a semantic-based system for exercise programming and dietary advice. In the system, users can specify muscles they want to develop.

Domains& Content: The exercise ontology focuses on the activity domain. It classifies exercises as antagonistic, assistant, main or secondary, assigns them into groups, and arranges them in a specific order. Exercises are also classified as beginner, intermediate or advanced. Different exercise types ("named exercises") are described. Some profile information is given regarding the level of experience needed for an exercise (beginner, intermediate, advanced).

Development: The ontology is not comprehensive and has only been developed to the point where its usefulness for the system can be shown.

Implementation and Validation: The system was verified experimentally by being tested for multiple usage scenarios.

Health and Meal Ontology (70)

Aim: This ontology was created in the context of a semantic cross-domain framework for designing healthcare mobile applications to generate recommendations of training and nutrition planning. It focuses on supporting runners in creating meal and workout plans.

Domains& Content: The health and meal ontology consist of information on nutrition and training plans. It covers the profile, activity and temporal domains. For the profile, the ontology contains general profile information (not further specified), clinical diagnoses and physical characteristics such as body mass and fitness level (based on a test within the developed system). For activities, the ontology contains a list of exercises that are split into different types such as endurance_work, physical_strength and warm_up, and recess. The effect of the exercises is also integrated. For the time domain, duration of exercises is mapped, as well as days.

Development: There is no information on the development of the ontology in the available documentation.

Implementation and Validation: The generateed training and meal plans are validated by a panel of 100 specialists and users.

Mining Minds Context Ontology (66,67)

Aim: The Mining Minds Context Ontology was created as part of a system for behaviour identification. The ontology enables us to infer high-level context from low-level information. The ontology is freely available online.

Domains& Content: The ontology covers the profile, activity, location and time domains. For the profile domain, the Mining Minds Context Ontology contains disease risks and physical characteristics such as blood pressure. On top of that, it contains emotional states as a psychological factor. For the activity domain, it contains a list of activities (non-physical and physical activities) and categorizes them based on intensities and context domains. The physical location can be determined based on a pre-existing list and a start- and end-time can be mapped for each activity. The main concept of the ontology is the Context class which defines different high-level context. The low-level information is modelled by the Activity, Location and Emotion class.

Development: There is no information on the development of the ontology in the available documentation.

Implementation and Validation: The Mining Minds Ontology has been proved to be flexible enough to operate in real life scenarios in which emotion recognition systems may not always be availabe. The ontology has been evaluated in terms of robustness using pellet.

Ontology for assessing PA and SB (78)

Aim: The aim of this ontology was to support more accurate assessments of physical activity and sedentary behavior.

Domains Content: The proposed ontology characterizes PA and SB and the context in which it occurs. It covers all domains that are discussed in this review.

For the profile domain, the ontology covers sociodemographic information such as age, education, occupation and cultural background, as well as physical characteristics such as bodyfat, body mass, height, and blood pressure. Lastly, the ontology contains psychological characteristics. The ontology contains a list of potential activities, different intensities and equipment. The context of the activity is described in terms of the location using GPS, but also categorizing different building and whether an activity takes place inside or outside. Lighting, weather and temperature are also taken into account. Usability, accessibility and supervision of a given location can also be mapped. For the time, duration and start-time are included, as well as the day. For the social context, social purpose and whether or not an activity was done in group are included. Relationship were integrated based on the Friend of a friend ontology.

Development: Existing ontologies, such as the Friend of A Friend ontology or the Relationship ontology, were reused. For physical activities, the ontology bases itself on the Compendium of Physical Activities.

Implementation and Validation: Information on implementation and validation were not present in the available documentation.

The Ubiquitous Exercise Plan Generation for Personalized Fitness (75,76)

Aim: The Ubiquitous Exercise Plan Generation for Personalized Fitness (UFIT) aims to provide personalized exercise plans.

Domains& Content: UFIT incorporates information on the profile, activity and time domain.

The plans are based on user's profile and health status, incorporating the Health Level Seven International (HL7) data on physical fitness and health screening. Users are described in terms of age and sex, existing diagnoses, preferences and current lifestyle, current fitness level and health indicators such as blood pressure or blood glucose.

Physical activities are described in terms of activity type, intensity and effect of exercises. In addition, starting time, duration and frequency can be described.

Development: The researchers conducted a requirement study with field experts for the development of their system. The ontology was developed following the Ontology Development 101 procedure. It is constituted by the exercise, user profile and health screening ontologies. A problem-oriented medical record ontology HL7-sample-plus-owl was reused in constructing these ontologies.

Implementation and Validation: The ontology was evaluated using competency questions. It was also implemented within the UFIT system which was evaluated using usage scenarios and case studies considering usability, as well as questionnaires.

Ontology of Motivational Messages (68)

Aim: The ontology of motivational messages for physical activity coaching models the message intention and components, but also its content. This way, messages can be categorized into multiple classes and be retrieved according to the needs of the user. The ontology is supported by categorization and retrieval methods for coaching in the domain of physical activity.

Content: The ontology covers information on the activity, location and time domain. Activities are described in terms of types (including non-PA types of activities), intensity and needed elements such as equipment or pets. Information on the physical context included a list of locations and the distinction between indoor and outdoor. For the time, the moment of the day (morning, afternoon etc) is included.

Development: There is no information on the development of the ontology in the available documentation.

Implementation and Validation: There is no information on the validation of the ontology in the available documentation.

Nuadu Ontology Collection (81)

Aim: The Nuadu Ontology collection has been created as a new approach for collecting and sharing personal health and wellness information based on a Personal Health Record. The formal upper ontology combines a set of domain ontologies representing different aspects of personal health and wellness, with some of them relevant to this review.

Content: The Nuadu Ontology Collection contains information on the profile, activity, location and time domain.

For the profile domain, a general profile contains an identifier and sociodemographic information such as age, sex and occupation. On top of that, current clinical diagnoses, lifestyle or habits, fitness level, objectives and preferences are included. Lastly, some health indicators such as blood pressure were included. Activities are described in terms of type and intensity. Location is described based on a list of available environments / locations. Time is described in terms of the duration of the activity, and specific events taking place.

Development: There is no information on the development of the ontology in the available documentation.

Implementation and Validation: There is no information on the validation of the ontology in the available documentation.

Patient Domain Ontology (69)

Aim: The patient domain ontology was developed as part of an approach for follow-up assessment for continuous and personalized chronic disease management. The system aims to provide clinical decision support during assessments of chronically ill patients at home. It should be able to analyze data, address abnormal conditions, provide lifestyle recommendations, provide instructions on medications, summarize results and customize management plans.

Content: The ontology contains information on the profile and activity domains. The profile domain contains sociodemographic information such as age and sex, information on current treatment (medication), diagnoses and health risks, physical characteristics such as height, body mass, body weight and health indicators such as blood pressure, and lastly preferences regarding physical activity. Activities are described in terms of their type, intensity and possible contraindications.

Development: For development, the authors followed the steps of Knowledge acquisition which results in a semi-formal ontology metamodel. The metamodel is then manually transformed into a formal ontology format. The framework was instantiated using real data from 115477 follow-up assessment record.

Implementation and Validation: The system was evaluated in the case study of a type 2 diabetic patient follow-up assessment. Using historical data as the gold standard, the system achieves a rate of 99.93% accuracy and 95% completeness.

Semantic Mining of Activity, Social, and Health data (56)

Aim: The SMASH ontology was developed as part of an ontology-based deep learning model (ORBM+) for human behavior prediction over undirected and nodes-attributed graphs. The aim of the system is to understand and predict individual behaviour in relation to the social communities around it.

Content: The core concepts of the three modules in the SMASh system are social networks, physical activity and biomarker measures. It covers the profile, activity and social domain, with a clear focus on the social domain. For the profile domain, SMASH contains sociodemographic descriptors such as age, sex, education, ethnicity and occupation as well as the address. On top of that, SMASH contains physical characteristics such as height, body mass, body fat, and health indicators such as blood pressure and other health-related risk factors. For the activity domain, SMASH contains a list of physical activities. For the social domain, SMASH can describe social interactions, relationships, on- and offline events as well as social communities.

Development: SMASH (Semantic Mining of Activity, Social, and Health data) was created top-down and later validated and refined using data-driven bottom-up techniques.

Implementation and Validation: The ORBM+ model has tested experimentally on both real and synthetic health social networks and has been more effective compared with conventional methods.

Extensible Context Ontology for Persuasive Physical-Activity Applications (65)

Aim: The goal of the Extensible Context Ontology for Persuasive Physical-Activity Applications (ECOPPA) is to provide a formal context modeling scheme for applications that promote physical activity.

Content: ECOPPA covers the profile, activity, location and time domain. Profile information is included in much detail, including identifiers, sociodemographic information such as age, sex and occupation, activity levels, psychological factors, physiological states, goals, preferences and fitness levels. A distinction was made between static and dynamic factors. For the activity domain, ECOPPA contains a list of physical and non-physical activities. Physical and sedentary activities in particular are extended from the activity concept. Activities can be described in terms of intensity. For the location domain, ECOPPA can determine location based on a provided list as well as based on GPS. On top of that, ECOPPA describes further environmental factors such as temperature, weather and lighting. Lastly, time can be described using start- and end-time as well as the duration of a given activity.

Development: There is no information on the development of the ontology in the available documentation.

Implementation and Validation: The ontology has been evaluated in terms of reasoning performance, which was found to increase almost linearly with respect to the number of instances.

Ontology of Physical Exercises (54)

Aim: The Ontology of Physical Exercises (OPE) provides a template for describing physical exercise.

Content: It covers exclusively the activity domain, but does so in much detail. Exercises can be described in term of its component movements, engaged musculoskeletal parts, related equipment or monitoring devices, intended health outcomes, and target ailments for which the exercise might be employed as a treatment or preventative measure.

Development: There is no information on the development of the ontology in the available documentation.

Implementation and Validation: The ontology was evaluated using interviews with domain experts and web developers.

ActivO (43)

Aim: The ActivO ontology is bared of COSAR, a hybrid reasoning system for context-aware activity recognition. Its focus lies on recognizing activities based on location.

Content: The ActivO ontology contains information on the activity, location, temporal and social domain. For the activity domain, it contains a list of (physical and non-physical) activities, divided by domain. The social domain is also included in so far as that one of the domains is contains social activities which are defined as having two or more actors. Some specific social interactions, such as talking, are also integrated. For the location domain, it contains an extensive list of possible locations that are connected to each other (e.g. bathroom is in a house) and are further described using attributes (e.g. bathroom has sink). ActivO also makes a distinction between indoor and outdoor locations, and for outdoor locations also between pedestrian and non-pedestrian locations. For the temporal domain, the duration of an activity can be taken into account.

Development: There is no information on the development of the ontology in the available documentation.

Implementation and Validation: The ontology was implemented into the COSAR system and validated experimentally using real data. The system showed great computational efficiency and accuracy.

Ontology Model for Diabetes Patients (43)

Aim: The Ontology Model for Diabetes Patients (OMDP) aims to be a comprehensive framework for diabetic patients to resolve the ambiguity and semantic inconsistency in current discourse and provide support for clinical diagnosis.

Content: The OMDP contains information on the profile and activity domains. The profile domain extensively focuses on medical information, such as current treatment and medication, diagnoses, disease risks and family history, blood pressure and blood glucose levels, as well as physical characteristics such as body mass. Some general sociodemographic information, such as age and sex, is also included. The general profile is not further described in the documentation, but seems to be more extensive. For the activity domain, OMDP includes a list of (physical and non-physical) activities. This list also contains the instance "exercise" at different intensities (light, moderate, strenuous).

Development: For development, the OMDP follows four phases, namely the OMDP preparation stage, OMDP class initialization stage, merging top-level ontology, and OMDP evaluation stage. Throughout the development, existing ontologies were re-used and domain experts were involved.

Implementation and Validation: The OMDP's logical consistency was assessed by the Pellet Reasoner, and its content coverage was evaluated by experts. OMDP was implemented into the system and validated experimentally regarding reusability, integrality and performance aspects such as accuracy, specificity and sensitivity of treatment recommendations.
